# Unveiling Promising Modalities and Enhancing Patient Outcomes in Graves’ Disease Treatment: A Systematic Review and Meta-Analysis

**DOI:** 10.7759/cureus.60829

**Published:** 2024-05-22

**Authors:** Hadeel Almutairi, Faisal S Alqadi, Rama K Alsulaim, Ghada Y AlKhoraiji, Lana Alwasel, Latifh M Alharbi, Layan A Alharbi, Samar Alsamiri, Betool R Alqfari, Taif A Almayouf, Flora Alrumaih, Fakhri M Almutairi

**Affiliations:** 1 Department of Surgery, Unaizah College of Medicine and Medical Sciences, Qassim University, Unaizah, SAU; 2 Medicine, College of Medicine, King Khalid University, Abha, SAU; 3 College of Medicine, Umm Al Qura University, Al Qunfudhah, SAU; 4 Department of Surgery, Qassim University, Unaizah, SAU; 5 Diabetes and Endocrinology, Al-Rass General Hospital, Ar Rass, SAU

**Keywords:** graves' diseas, graves relapse, including graves’ disease (gd), auto immune, graves’s diseases

## Abstract

Graves' disease (GD) is an autoimmune condition of the thyroid. The hyperthyroidism manifested by patients affected by this disease is caused by the production of autoantibodies against the thyroid-stimulating hormone (TSH, or thyrotropin) receptor (TSHR), which mimic the effects of the hormone on thyroid cells, thereby stimulating autonomic production of thyroxine and triiodothyronine. Deciding on a therapeutic approach to this condition presents intricate dilemmas for both clinicians and patients. Each of the three available treatment modalities is grounded in evidence-based medicine, affirming its efficacy. This systematic review and meta-analysis aimed to assess the effect of carbimazole (CBM), radioactive iodine (RAI), and surgery in treating GD and provide evidence-based recommendations for healthcare providers regarding the optimal management of the condition based on a comprehensive analysis of effectiveness, safety, patient satisfaction, and recovery outcomes.

This systematic review and meta-analysis adhered to the Preferred Reporting Items for Systematic Reviews and Meta-Analyses (PRISMA) guidelines. We used the PubMed and Google Scholar databases to conduct a thorough web search for articles published between January 2019 and September 2023. The meta-analysis was carried out using Resource Manager (Revman) 5.4.1. The study found that propylthiouracil (PTU) or methimazole/carbimazole (MMI/CBM) treatment increases the risk of hyperlipidemia in patients with hyperthyroidism. Once in a euthyroid state, glucose tolerance increases; for children with GD, a computer model for customized dosing has been created. To sum up, CBM, surgery, and RAI are all useful treatment options for GD. Using steroids in conjunction with radiation therapy may help prevent Graves' ophthalmopathy (GO).

## Introduction and background

Graves' disease (GD) is an autoimmune condition of the thyroid gland. Hyperthyroidism in patients with this disease is attributed to the production of autoantibodies against the thyroid-stimulating hormone (TSH, or thyrotropin) receptor (TSHR), which mimic the effects of the hormone on thyroid cells, resulting in the stimulation of autonomic production of thyroxine and triiodothyronine [1]. The annual incidence of GD is 16 cases per 100,000 women and three cases per 100,000 men, and the usual age of occurrence is between 30 and 60 years [2]. Timely intervention for overt hyperthyroidism is crucial, as leaving it untreated can lead to heightened morbidity and mortality [3], primarily due to cardiovascular issues such as pulmonary hypertension, stroke, heart failure, atrial fibrillation, and angina pectoris [4], as well as skeletal complications like osteoporosis. Moreover, GD adversely affects the long-term quality of life, either due to the disease's progression or its therapeutic interventions [3].

The primary objective in the management of GD is to regulate and rectify the disorder, grounding the treatment strategy in an understanding of GD's pathophysiological mechanisms, particularly the antigen-antibody interactions within the thyroid glands. The selection of treatment for GD is influenced by various factors, including the intensity of thyrotoxicosis, ophthalmopathy, patient age, the size of the goiter, the accessibility of treatment options, treatment outcomes, and the presence of other associated health conditions [5]. Treatment options encompass antithyroid drugs (ATDs, thionamide), surgical intervention, and radioactive iodine (RAI) therapy [6]. Choosing a therapeutic approach presents intricate dilemmas for both clinicians and patients. Each of the three available treatment modalities is grounded in evidence-based medicine, affirming its efficacy. Gaining insights into the pros and cons of each available choice will enhance the decision-making process and ensure a more informed and balanced strategy. In light of this, we performed this systematic review and meta-analysis to assess the effect of carbimazole (CBM), RAI, and surgery in treating GD and provide evidence-based recommendations for healthcare providers regarding the optimal management of GD based on a comprehensive analysis of the effectiveness, safety, patient satisfaction, and recovery outcomes.

## Review

Methods

Literature Search Strategy

This systematic review and meta-analysis was conducted following the Preferred Reporting Items for Systematic Review and Meta-Analysis (PRISMA) guidelines. A broad electronic search was conducted through PubMed and Google Scholar databases for studies published between January 2019 and September 2023, by using the following terms: Carbimazole OR Radioactive Iodine OR Thyroidectomy AND Graves’ disease.

Inclusion and Exclusion Criteria

We included all relevant studies published between January 2019 and September 2023, such as randomized control trials, retrospective cohort studies, clinical trials, prospective randomized clinical trials with patients diagnosed with GD, studies that used different treatment methods for GD, and studies written in English. Duplicates, studies with a high potential for bias, and studies that did not fit our requirements were excluded. Furthermore, all other types of studies and those published in other languages were excluded.

Selection of Articles and Data Extraction

All articles resulting from the primary search were imported to Mendeley for duplication removal. The results after deduplication were imported to Rayyan and independently screened by four authors (F.A.R., L.A.W., S.A.S., and B.R.Q.) based on titles and abstracts. The same four authors reviewed the full text of all studies. Disagreements at any step of the screening process were handled through debate and consensus among all authors. The four authors independently extracted the data. It included the name of the authors, year of publication, city, study design, sample size, inclusion and exclusion criteria, age, technique of treatment, outcomes, result, reference number, and comments. For every study or trial that was included, two reviewers assessed the risk of bias independently and discrepancies were settled by consensus and discussion.

Statistical Analysis

The data were analyzed using SPSS Statistics version 28 (IBM Corp., Armonk, NY). The heterogeneity of the research was assessed by using the Q test and the I^2^ statistic. Forest plots with 95% confidence intervals (CIs) were used to display the data. Odds ratios (ORs) or standardized mean differences (SMDs) with 95% CIs were computed for both continuous and categorical outcomes. Sensitivity analyses and funnel plots were used to assess the robustness of the results and the existence of publication bias, respectively. A significance level of 0.05 was set for all analyses.

Results

**Figure 1:**
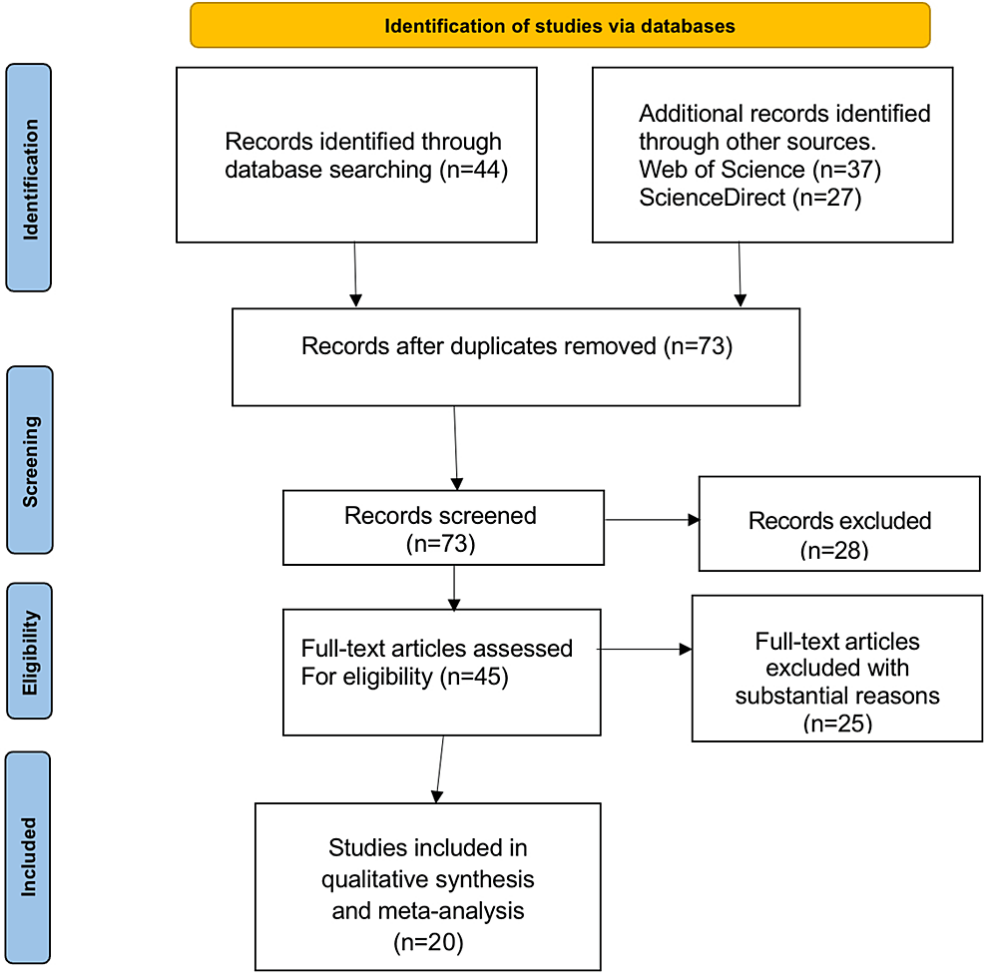
PRISMA flow diagram PRISMA: Preferred Reporting Items for Systematic Review and Meta-Analysis

As shown in the PRISMA flow diagram in Figure 1 below, a thorough search of the aforementioned databases initially yielded 108 studies that met our search criteria. After 35 duplicate studies were removed, 73 publications remained for additional screening. Additionally, 28 records were discarded as their design did not satisfy our criteria, leaving 45 articles for eligibility review. However, after a review by two independent senior researchers, 25 of these studies were discarded for failing to meet the predetermined standards. Twenty publications that satisfied the requirements deemed appropriate for the systematic review were ultimately included,

Study Characteristics

All 20 studies were in English and included retrospective and prospective cohort studies, clinical trials, and randomized trials. Furthermore, the studies were conducted in various countries/geographical areas. There was a significant variation among the studies in terms of the year, location, sample size, design, inclusion criteria, and outcomes. A summary of the included studies is presented in Table 1.

**Table 1:** Summary of the included studies APAC: Asia-Pacific region; ATDs: antithyroid drugs; AZA: azathioprine; FT3: free triiodothyronine; FT4: free thyroxine; GD: Graves’ disease; GDd: Graves’ disease duration; GO: Graves’ ophthalmopathy; IVGC: intravenous glucocorticoids; LT-MMI: long-term methimazole; LiCO_3_: lithium carbonate; ML-OD: medial plus lateral wall orbital decompression; MMI: methimazole; OD: orbital decompression; OGC: oral glucocorticoids; RAI: radioactive iodine; Se: selenium; TSH: thyroid-stimulating hormone; vitD: vitamin D

Author	Region	Sample size	Study design	Participant details	Results/outcomes and main findings
Gallo et al., 2022 [7]	Italy	42	Randomized, single-blinded, controlled, intervention trial	Caucasian patients with recently diagnosed GD between the ages of 18 and 70 years, in good health (apart from hyperthyroidism), and able to provide written informed consent had their levels of circulating calcidiol [25(OH)D, VitD] and Se measured. Distinctive uptake at a thyroid scan, a hypoechoic gland with increased vascularization at ultrasonography, elevated serum free thyroxine (FT4) and/or free triiodothyronine (FT3), and suppressed serum thyrotropin (TSH) and serum free thyroxine (FT4) and/or free triiodothyronine (FT3) levels were among the criteria (2) that were used to diagnose GD. The eligibility criteria were serum Se concentration of less than 120 mcg/liter and plasma VitD concentration of less than 30 ng/ml	According to the study's findings, which were based on a cohort of consecutively diagnosed GD patients with borderline low Se levels and VitD insufficiency, 1) Se and VitD supplementation helps restore euthyroidism during MMI treatment; and 2) Se and VitD supplementation enhances the improvement of quality of life during MMI treatment. If even a slight deficiency of these micronutrients is discovered, it is reasonable to suggest that Se and VitD status be evaluated at the time of GD diagnosis and that Se and VitD supplementation be provided at appropriate and safe dosages
Ramtahal and Dhanoo, 2016 [8]	Trinidad and Tobago	1	Case report	Intolerance to carbimazole	Effective
Vannucchi et al., 2019 [9]	Italy	121	Prospective RCT	Before RAI, 99 hyperthyroid patients with GDd <5 years and no prior inactive GO were randomized to receive IVGC (n = 49) or OGC (n = 50); 22 patients with GDd >5 did not receive steroids and were studied as controls	After RAI, no patient receiving prophylaxis experienced GO. One woman in the control group, who was not receiving steroid prophylaxis and whose TSH level was noticeably elevated, experienced a brief reactivation of GO, which resolved on its own when her euthyroidism was restored
Parameswaran et al., 2021 [10]	Asia-Pacific countries	542	Survey	Practicing physicians in APAC received an email with a link via their community specialty societies. An example of a patient was provided, who had moderate hyperthyroid symptoms for two months, a moderate goiter, and no indications of GO. Diagnoses, treatment options, and follow-up were the main topics of inquiry	Achieving a euthyroid state
Abbara et al., 2020 [11]	UK	441	Retrospective cohort study	Graves' disease-related hyperthyroidism was diagnosed by biochemical hyperthyroidism, positive autoantibody to the TSH receptor (>0.3 U/mL), or a technetium thyroid scan that was consistent with the disease	Both FT3 and FT4 decreased proportionately
Wu et al., 2020 [12]	Taiwan	13,667	Retrospective cohort study	-	The euthyroid state was achieved
Lakshmana Perumal et al., 2019 [13]	India	36	Observational study	Individuals on long-term steroid therapy, as well as those with known conditions such as malignancy, active infection, polycystic ovary syndrome, chronic kidney disease, hepatitis or cirrhosis, or prediabetes or diabetes mellitus	Stable euthyroid state
Raheem, 2021 [14]	Sri Lanka	89	Prospective cohort study	-	At six months, there was no difference in the outcome of 10 mCi radioactive iodine therapy between patients who had been off carbimazole for 3–7 days and 8–30 days. Removing carbimazole for more than seven days does not yield any additional benefits
Allam et al., 2023 [15]	Saudi Arabia, Egypt	270	A randomized controlled open-label clinical trial	The patients with extreme symptoms (heart rate >120 beats/min, hyperthyroid heart disease, or weight loss >10 kg) and/or levels of free T4 or free T3 exceeding two times the upper limit of normal (FT4 ≥3.6 ng/dl or FT3 ≥8.4 pg/ml, respectively) were included as cases of moderate and severe GD	AZA may be a cutting-edge, reasonably priced, safe, and effective medication that gives GD patients hope for an early and sustained medical remission
Sultana et al., 2021 [16]	-	75	Randomized clinical trial	1. Patients in the 16–50 age range who had just received a hyperthyroid diagnosis were treated with carbimazole. 2. Patients in the 16–50 age range who had recently been diagnosed with hyperthyroidism were administered a combination of carbimazole and antioxidants (at least for two months). 3. All genders	Supplementing with antioxidants may help decrease the oxidative damage caused by hyperthyroidism and may have a positive impact on the reduction of thyroid hormone levels
Alnajjar et al., 2023 [17]	Riyadh, Saudi Arabia	1	Case report	-	Severe neutropenia induced
Saadat et al., 2022 [18]	-	64	Randomized clinical trial	-	When it came to achieving euthyroidism more quickly and maintaining better control of hyperthyroidism over a 60-month follow-up period, LT-MMI treatment proved to be more effective than radioiodine in treating post-RAI relapses of hyperthyroidism
Levy et al., 2022 [19]	UK	56,741	Retrospective cohort	A median follow-up duration of 10.9 years was observed for the 1,479 patients who were included. Thyrotoxicosis had autoimmune (85.9%), nodular (9.1%), and mixed (5.0%) etiologies. Antithyroid medications (ATDs), thyroidectomy, and radioiodine (RAI; 555 MBq fixed dose) were the treatment modalities used	By creating a long-term treatment plan that consists of antithyroid drugs (ATDs), radioiodine (RAI; 555 MBq fixed dose), and thyroidectomy
Steffens et al., 2023 [20]	Switzerland	44	Retrospective cohort	-	A computer model for pharmacometrics was created using FT4 measurements, carbimazole, or carbimazole and levothyroxine dosages, which included two clinically significant covariate effects: the severity of the disease and the patient's age at diagnosis
Delshad et al., 2020 [21]	-	195	Retrospective	Comprised children and teenagers diagnosed with GD before the age of 19 years between January 2000 and October 2020	The length of ATD treatment was associated with an increase in the cumulative remission rate. Before receiving a definitive course of treatment, children and adolescents with GD disease may benefit from long-term MMI treatment
Pereira et al., 2022 [22]	-	42	Prospective cohort study	-	Following OD, both surgical techniques resulted in significant improvements in clinical and radiological exophthalmometry; however, balanced OD produced the greatest improvement
Thamcharoenvipas et al., 2019 [23]	-	60	Randomized clinical trial	Weight >50 grams of the thyroid gland and age >18 years were the inclusion criteria	Twelve months later, group A's cure rate was 45% (9/20), while group B's was 60% (12/20), and group C's was 80% (16/20). Between groups C and A, the mean percentage of cases cured at 12 months varied by 35% (7.0 to 66.8%); p = 0.02. After adjusting for sex, there was a statistically significant difference in the cure rates between groups C and A throughout the follow-up (adjusted OR = 3.09; 95% CI = 1.32–7.20; p =0.009), but there was no significant difference between groups B and A or C and B in terms of the primary and/or secondary efficacy endpoints. One group in C, four in A, and two in B experienced adverse effects from the treatment, accounting for 12% (7/60) of the total. Most likely, LiCO_3_ side effects were the cause of four of them
Azizi et al., 2019 [24]	Iran	66	Randomized trial	The following criteria were considered for inclusion: free thyroxine (FT4), thyrotropin, 0.4 mU/L, age 18 years, and an absence of any indication of comorbidity in the past	Methimazole therapy was administered for 120 months with no other adverse events noted, except for 3 cases of cutaneous reactions
Xu et al., 2019 [25]	China	103	-	For inclusion, the following conditions had to be met: 1) not having a history of using ATD or being contraindicated for ATD treatment before admission, and 2) having met the Chinese Diagnosis and Treatment of Thyroid Diseases diagnostic criteria for GD. Three things were necessary for patients to have: 3) no coexisting diseases such as heart, liver, or kidney failure; 4) low free thyroxine (FT4) and TSH; and 5) careful follow-up with medical advice	Utilizing MMI and Se together may help patients' thyroid activity, which could lead to an efficient treatment for GD in clinical settings
Leite et al., 2021 [26]	Sau Paulo, Brazil	42	Randomized prospective comparative study	The following criteria were used for inclusion: 1) the diagnosis of GO in the inactive phase; 2) the provision of written informed consent; 3) age >21 years; 4) euthyroidism; 5) exophthalmometry >20 mm; 6) the absence of abnormalities in the eyes, such as degenerative myopia, microphthalmos, and anophthalmia; 7) the absence of other abnormalities in the orbit, such as congenital defects and prior fractures; 8) the ability to cooperate with study procedures; 9) the capacity to adhere to the consultation schedule; and 10) the absence of contraindications for OD in the preoperative clinical evaluation	Both groups showed statistically significant reductions in exophthalmometry (p = 0.001), with the ML-OD group showing a higher reduction (p = 0.010)

Risk of Bias Analysis

**Figure 2:**
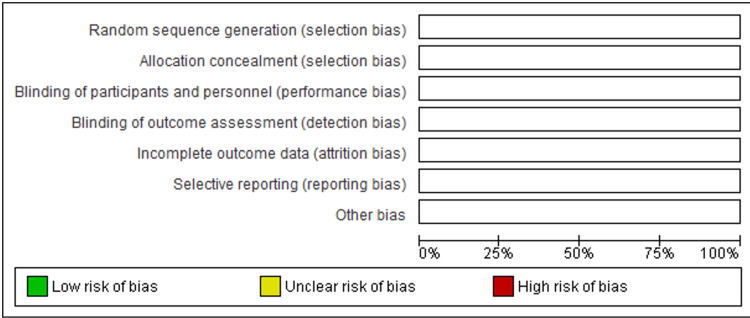
Risk of bias analysis

As illustrated in Figure 2 below, none of the included studies showed any risk-related biases, such as incomplete outcome data, selective reporting, participant and staff blinding, random sequence generation, allocation concealment, and other kinds of biases.

Forest Plot of Recovery Time

**Figure 3:**
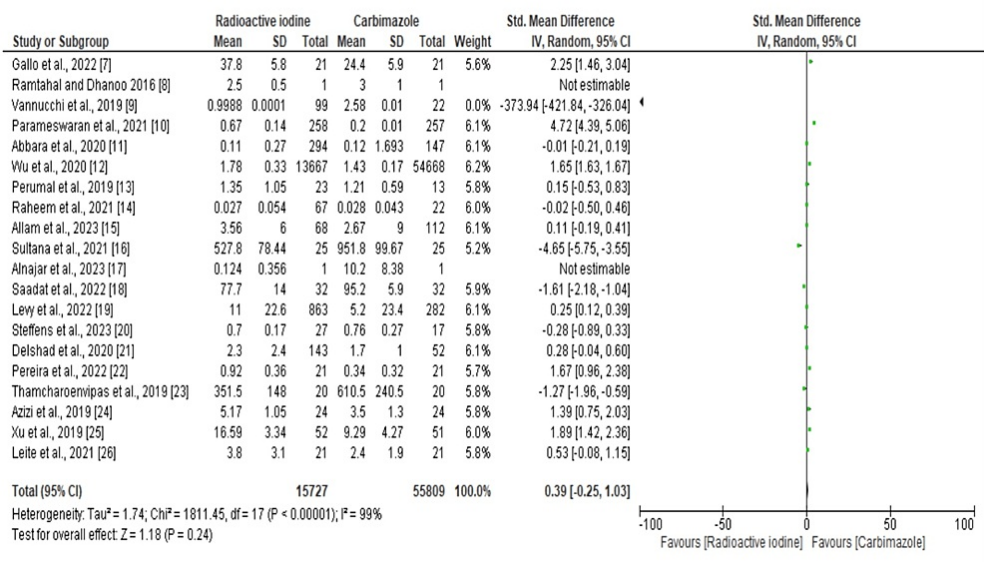
Forest plot of recovery time

Twenty studies totaling 72579 participants were included in the meta-analysis. The sample sizes ranged from 1 to 54,668. However, some participants left the study, which resulted in a reduction in the total sample size to 71,536. Significant heterogeneity was evident across all studies, according to the forest plot results (Figure 3) (SMD: -0.39, 95% CI: -1.25 to 1.03; I^2^ = 99%). However, the overall effect of the two doses did not differ significantly with regard to patients’ recovery time.

Forest Plot of the Effectiveness of the Methods

**Figure 4:**
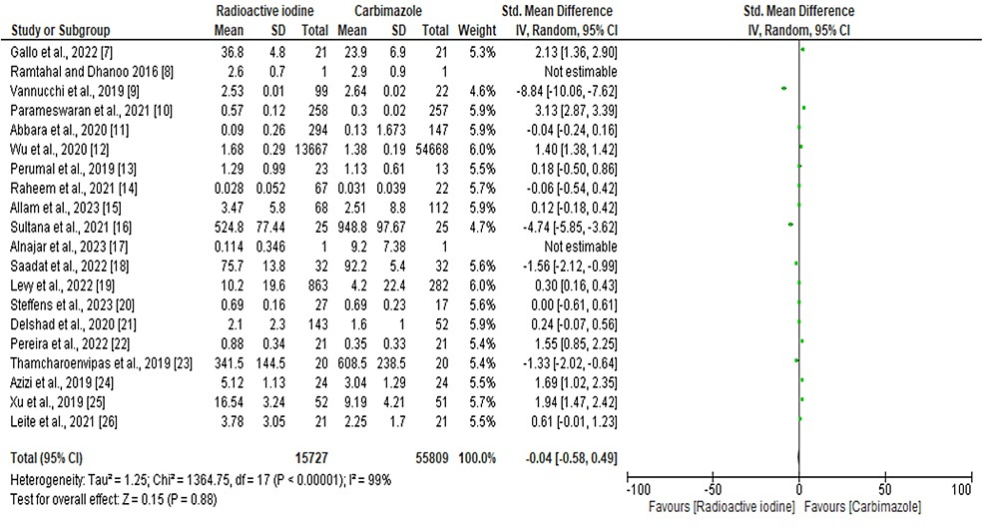
Forest plot of the effectiveness of the methods

As illustrated in Figure 4, a statistically significant level of heterogeneity was observed across the studies. The I^2^ value was 99%. RE: 0.04, 95% CI: -0.58 to 0.49, p = 0.00001. However, the overall effect did not vary significantly across the studies.

Funnel Plot

**Figure 5:**
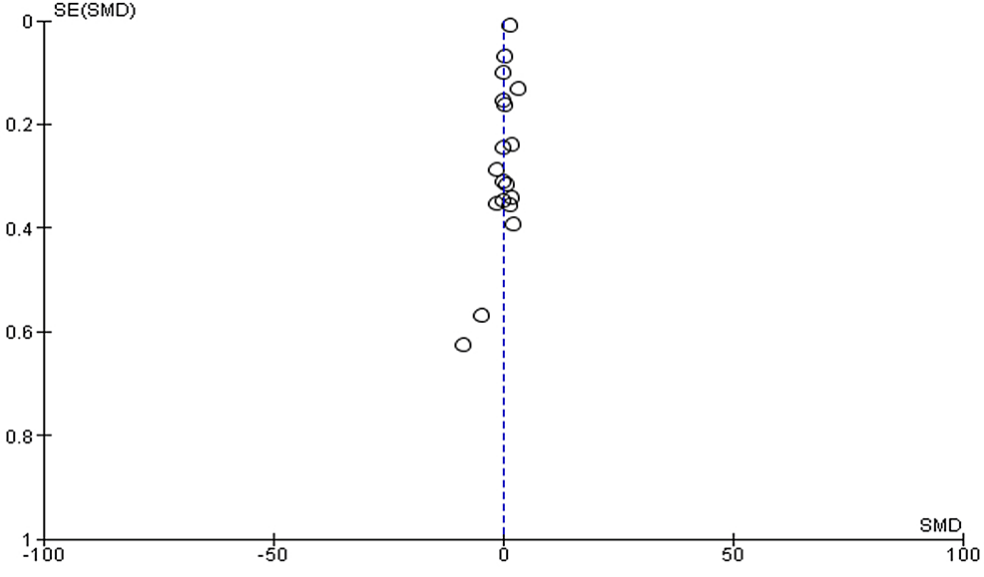
Funnel plot depicting publication bias

An analysis of funnel plots revealed an asymmetrical funnel. Figure 5 illustrates the relevant results, which suggested the possibility of bias on both sides, primarily to the right.

Discussion

This systematic review and meta-analysis focused on assessing the effect of carbimazole, methimazole, RAI, and surgery in treating GD and providing evidence-based recommendations for healthcare providers regarding the optimal management of GD based on a comprehensive analysis of effectiveness, safety, patient satisfaction, and recovery outcomes. Our findings reveal that the intervention group demonstrated a greater improvement than the MMI group when comparing the ThyPRO composite score at 45 days (-14.6, 95% CI: -18.8 to -10.4), 180 days (-9, 95% CI: -13.9 to -4.2), and 270 days (-14.3, 95% CI: -19.5 to -9.1) (respectively, -5.2, 95% CI: -9.5 to -1; -5.4, 95% CI: -10.6 to -0.2 and -3.5, 95% CI: -9 to -2.1, 0-6 months and 6-9 months, p<0.05). The results suggest that reaching optimal levels improves the early efficacy of MMI treatment when serum and vitamin D levels are suboptimal [7]. Based on the findings, subclinical hypothyroidism was determined based on elevated TSH levels, normal free thyroxine (T4) levels, and positive thyroid peroxidase antibody levels. Levothyroxine was used to treat the patients successfully, allowing the blood pressure to return to normal without needing antihypertensive medication [8]. The findings of the study also reveal that steroids can prevent the risk of RAI-induced GO in all patients with GDd <5 years. Patients with GDd >5 years may not require such treatment.

The preventive effect of steroids may be connected to the blunting of TRAb elevation following RAI [9]. In addition, the study reveals that antithyroid drug therapy (79%) with MMI or carbimazole is the recommended first-line treatment. RAI (19%) and surgery (2%), in that order, are the next most common treatments. A third of the participants indicated that they preferred a subtotal thyroidectomy over a total one in the event of surgery. ATDs are still the recommended treatment for mild Graves’ orbitopathy (GO), with 67% of clinicians preferring them over surgery (20%). The recommended course of treatment for a patient intending to become pregnant stayed the same; however, during the first trimester, propylthiouracil (PTU) emerged as the preferred ATD agent [10].

TSH receptor antibody titers were positively correlated with baseline thyroid hormone levels (p<0.0001). In the hyperthyroid state, baseline free triiodothyronine (fT3) was linearly correlated with free thyroxine (fT4) levels (fT3 = fT4*0.97-11), and it decreased proportionately with carbimazole. The daily percentage declines in fT4 and fT3 were correlated with the dosage of carbimazole (p<0.0001). Following the same dosage of CBM, the follow-up visit saw a smaller decline in thyroid hormones than the initiation visit [11]. Furthermore, in patients with hyperthyroidism, the overall incidence of hyperlipidemia was significantly higher (18.7 vs. 11.8 cases/1,000 persons-years; adjusted HR: 1.5; 95% CI: 1.41-1.59). Patients with hyperthyroidism demonstrated a 1.78-fold (95% CI: 1.50-2.11) and 1.43-fold (95% CI: 1.27-1.60) higher risk of hyperlipidemia with only PTU or MMI/CBM treatment, respectively, compared to those without hyperthyroidism. Furthermore, patients with hyperthyroidism undergoing surgery alone or in conjunction with I131 therapy tended to be at higher risk for developing hyperlipidemia. PTU and MMI treatment increased the expression of lipogenic genes in hepatic cells while decreasing the expression of genes encoding circulating remnant lipoproteins [12].

Our results also noted that, at baseline, the glucose tolerance of two-thirds of the patients was abnormal. During follow-up, 34.7% of individuals with abnormal glucose tolerance at baseline continued to show abnormalities. The insulin resistance indices did not significantly change during the follow-up period. At baseline, the ISSI-2 index was significantly lower in patients with abnormal glucose tolerance, and it improved after reaching a euthyroid state [13]. However, based on the results, the study found that to account for inter-individual disease progression and treatment response in children and adolescents with GD, there is a need to present a customized pharmacometrics computer model that can describe individual FT4 dynamics under both CBM monotherapy and CBM/levothyroxine block-and-replace therapy. A computer model that is both predictive and clinically useful could improve and simplify tailored medication for children with GD, minimizing over- and under-dosing and averting unfavorable short- and long-term effects. For pediatric GD and other rare pediatric diseases, prospective randomized validation trials are necessary to further validate and optimize computer-supported personalized dosing [14].

According to the study, the azathioprine (AZA)1 and AZA2 groups had a higher rate of remission (87.5% and 87.5% vs. 33.4%, p = 0.002) than the controls. The treatment did not significantly differ between the AZA and control groups throughout follow-up, but there were significant differences in FT3, FT4, TSH, and TRAb [15]. Furthermore, the AZA2 group experienced a significantly faster decline in FT4, FT3, and TRAb concentrations than the AZA1 group. During the 12-month follow-up, the control group experienced an insignificantly higher rate of relapse (10, 4.4, and 4.4%, p = 0.05, respectively) than did either the AZA1 or AZA2 group [16]. The MDA level was found to be significantly higher in the study groups (p<0.001) among hyperthyroid patients treated with CBM alone (Group B, 2.79 0.58 umol/L) compared to those treated with CBM and antioxidants combined (Group C, 1.57 0.29 umol/L) [17]. In cases of hyperthyroidism, it is crucial to keep patients in a long-term euthyroid state to reduce autoimmunity and hyperthyroid relapse. This frequently necessitates long-term CBM use. However, hepatotoxicity and severe neutropenia are uncommon but dangerous side effects of CBM [18]. It was also noted that LT-MMI treatment produced euthyroidism more quickly and maintained hyperthyroidism better over a 60-month follow-up period; it was superior to radioiodine in patients with post-RAI relapses of hyperthyroidism [19].

The study also revealed that the cornerstone of the treatment for juvenile GD is antithyroid medication, with long-term MMI therapy contributing to a higher rate of remission in children with GD [20]. Although both inferomedial and balanced OD effectively increased orbit capacity, the latter was more effective in lowering exophthalmos, most likely as a result of including the lateral wall. Following OD, both upper and lower eyelid retraction improved, but only lower eyelid elevation was associated with a decrease in exophthalmos [21]. For the treatment of juvenile GD, LT-MMI for 96 to 120 months is both safe and effective [22]. When using LT-MMI, the four-year cure rate for hyperthyroidism is nearly three times higher than when using ST-MMI treatment [23]. We propose that using MMI and Se together may help patients' thyroid activity, which could lead to an efficient treatment for GD in clinical settings [24]. When it comes to new-onset strabismus, IM-OD is just as safe as ML-OD. It is a good option for patients who do not need a significant reduction in exophthalmos [25]. More exophthalmos reduction and a more seamless postoperative recovery are provided by ML-OD. New-onset esotropia is possible in patients with preoperatively enlarged medial rectus muscles on CT, and preoperative esotropia is likely to worsen following OD [26].

This study has a few limitations. We excluded non-English studies, reducing the number of papers we examined. Another limitation was that while we were able to examine the outcomes of each trial, the outcomes differed between them. The small number of studies included in this study was a disadvantage in terms of conducting a meta-analysis due to the heterogeneity of the results. Furthermore, only the carbimazole/methimazole studies provided adequate data with comparable findings (cure after radioiodine treatment) to support a meta-analysis. Therefore, the findings of this study cannot be generalized for GD management in the general population.

## Conclusions

The thorough examination of several studies has led us to conclude that CBM is a useful therapeutic option for GD, improving symptoms and thyroid hormone levels. In addition to CBM, other effective treatment options include surgery and RAI, though their advantages and disadvantages may vary. Patients receiving RAI therapy may benefit from the use of steroids to prevent the development of Graves' ophthalmopathy. For GD, MMI/CBM-based antithyroid medication therapy is the recommended initial course of treatment; RAI and surgery are thought to be the backup plans. Customized dosages using pharmacometric computer models may help children with GD respond better to treatment. Furthermore, individuals with hyperthyroidism may be more susceptible to hyperlipidemia and abnormal glucose tolerance; these metabolic abnormalities may disappear when the patient reaches euthyroid status.
